# Tailoring Subthalamic Nucleus Deep Brain Stimulation for Parkinson's Disease Using Evoked Resonant Neural Activity

**DOI:** 10.3389/fnhum.2020.00071

**Published:** 2020-02-28

**Authors:** Wesley Thevathasan, Nicholas C. Sinclair, Kristian J. Bulluss, Hugh J. McDermott

**Affiliations:** ^1^Bionics Institute, East Melbourne, VIC, Australia; ^2^Department of Neurology, The Royal Melbourne and Austin Hospitals, Melbourne, VIC, Australia; ^3^Department of Medicine, The University of Melbourne, Melbourne, VIC, Australia; ^4^Medical Bionics Department, The University of Melbourne, East Melbourne, VIC, Australia; ^5^Department of Neurosurgery, St Vincent's and Austin Hospitals, Melbourne, VIC, Australia; ^6^Department of Surgery, The University of Melbourne, Melbourne, VIC, Australia

**Keywords:** evoked resonant neural activity, deep brain stimulation, subthalamic nucleus, Parkinson's disease, biomarker

## Introduction

Deep brain stimulation (DBS) applied to the subthalamic nucleus (STN) can be a highly effective therapy for Parkinson's disease (PD); however, there are significant issues which limit its effectiveness, reliability, and tolerability. Inaccurate electrode implantation is common, which can reduce efficacy and cause side-effects due to the undesired activation of neighboring brain regions (Okun et al., 2005; Paek et al., 2013; Rolston et al., 2016). Consequently, in most centers patients are typically kept awake during implantation surgery so that clinical assessments can help determine whether the positioning of electrodes is acceptable (Chakrabarti et al., 2014). The programming of stimulation is also often suboptimally performed, and there are many examples of patients being suddenly liberated from poor movement when chronically applied settings are changed (Okun et al., 2005; Sommer et al., 2015). Programming also confers a high burden to patients and neurologists, especially in the era of directional electrodes, often requiring multiple sessions over several months to identify the most effective DBS settings (Cagnan et al., 2019). Moreover, once programmed, DBS is then applied invariantly without “adapting” to the real-time fluctuating needs of the patient (Little et al., 2013; Priori et al., 2013).

A realistic solution to these issues is to use neuronal signals recorded from DBS electrodes to guide accurate electrode implantation (ideally with patients under general anesthesia), to automate programming, and to act as a feedback signal for continuous “adaptive” control. To achieve these aims, such a neuronal biomarker would likely need to localize to the STN (preferably the dorsal motor region where DBS is usually most effective; Herzog et al., 2004), reflect patient state and therapeutic effects with a reasonable time resolution, and, crucially, be reliably detectable in all patients and conditions, including under general anesthesia.

Potential biomarkers that may fulfill these criteria for STN DBS in PD include measures from spontaneous neural activity, such as beta oscillations (Little and Brown, 2012; Priori et al., 2013). However, beta band (13–30 Hz) activity is typically of small magnitude (microvolts) and can be variable across patients (Giannicola et al., 2010), making it challenging to reliably record with high fidelity, particularly using implantable, miniaturized systems (Neumann et al., 2016). Evoked responses elicited by DBS pulses offer alternative biomarkers (Ashby et al., 2001; Baker et al., 2002; Walker et al., 2012; Gmel et al., 2015; Kent et al., 2015; Romeo et al., 2019), including the recently identified phenomenon of evoked resonant neural activity (ERNA) (Sinclair et al., 2018).

## ERNA

**Figure 1:**
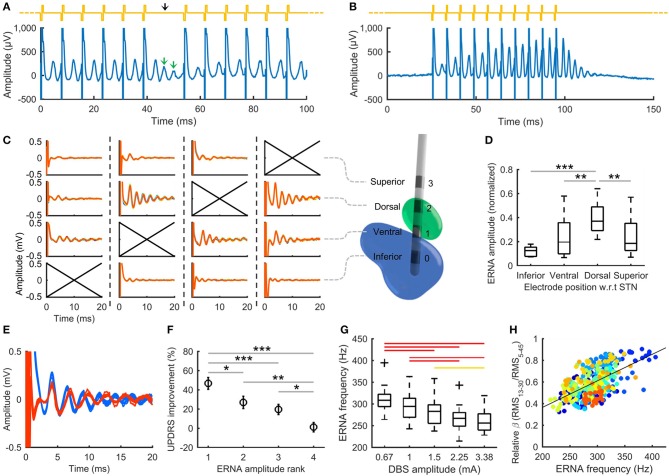
DBS evoked resonant neural activity (ERNA). **(A)** Periodically omitting one pulse in otherwise continuous 130 Hz DBS allows several ERNA peaks to be observed. Yellow trace: stimulation applied; black arrow: omitted pulse. **(B)** Short bursts of pulses (e.g., 10 pulses at 130 Hz) can be used as a “probe” to measure ERNA during periods off DBS therapy. **(C)** Applying burst probe stimulation in the vicinity of the STN elicits ERNA that varies with electrode position. Columns show the ERNA recorded at each electrode for different stimulating electrodes (indicated by crossed axes) in a single example STN from a person with PD. A 3D reconstruction illustrates the electrode positions (green: STN, blue: substantia nigra). **(D)** Normalized ERNA amplitude varies with electrode position with respect to (w.r.t) the STN in people with PD (20 hemispheres tested) (box: 25th−75th percentiles; line: median; whiskers: range). ***p ≤ 0.001, **p < 0.01, *p < 0.05. **(E)** ERNA recorded in a person with PD at electrode implantation (blue) and under general anesthesia 560 days postop (red). **(F)** Mean Unified Parkinson's Disease Rating Scale (UPDRS) improvement from stimulation after ranking electrodes within each hemisphere according to ERNA amplitude (rank 1: largest ERNA; bars: standard error). Results from 10 PD patients tested post-surgery (20 hemispheres). **(G)** ERNA frequency decreases with increasing DBS amplitude (19 hemispheres tested). Red bars: *p* ≤ 0.001; yellow bars: *p* < 0.05. **(H)** ERNA frequency correlates with relative beta band (13–30 Hz) amplitude across the stimulation levels shown in **(G)** (ρ = 0.58, *p* < 0.001). Colors represent different hemispheres tested. **(A,B,G,H)** reproduced from Sinclair et al. (2019), used with permission. **(C–F)** reproduced from Sinclair et al. (2018), used with permission.

ERNA is not a spontaneous signal like those detected in local field potentials, but rather is evoked by each DBS pulse applied in the vicinity of the STN. ERNA has a characteristic decaying oscillation waveform with the first peak typically seen around 4 ms after the DBS pulse (Figures 1A–C). ERNA has been found to be modulated by consecutive DBS pulses applied at therapeutic rates (e.g., 130 Hz) (Sinclair et al., 2018, 2019). As the duration of ERNA can extend well-beyond the time interval between pulses (7.7 ms for 130 Hz DBS), to appreciate multiple peaks of the decaying oscillation, a “window” needs to be created during otherwise continuous DBS, e.g., by periodically omitting an occasional pulse (Figure 1A). Occasionally omitting a pulse (e.g., once per second) is presumed to have a negligible impact on therapy. Alternatively, as stimulation is required to elicit ERNA, during periods off DBS therapy, an occasional burst of pulses (e.g., 10 pulses delivered at 130 Hz) can be used to “probe” for ERNA (Figure 1B). Such patterning of stimulation is not difficult to achieve from an engineering perspective, especially using devices designed to cycle stimulation on and off.

A multitude of parameters can be extracted from the ERNA waveform, including amplitude [e.g., root mean square (RMS) amplitude, or peak-peak amplitude] and frequency (e.g., latency between peaks). A key benefit of ERNA is its large amplitude, with the difference between the first peak and trough typically being hundreds of microvolts, orders of magnitude larger than spontaneous local field potentials. This large amplitude and the inherent time-locking of responses to stimuli, makes ERNA readily recordable using fully-implantable hardware, e.g., such evoked signals have long been employed for use in cochlear implants (Shallop et al., 1999).

ERNA has also been confirmed to be of neural origin and not an inadvertent artifact from stimulation (Sinclair et al., 2019a). Variation in ERNA amplitude with electrode position relative to the STN (Figures 1C,D) and its absence in recordings obtained from neighboring brain region (e.g., ventral intermediate nucleus, posterior subthalamic area) using the same system (Sinclair et al., 2018), provides clear evidence that it is not produced by the stimulation and recording hardware. The ERNA waveform is also not inverted by reversing the polarity of stimulus pulses, indicating that it has a neural basis (Sinclair et al., 2019a). Furthermore, the existence of ERNA has been corroborated by other, independent, research groups (e.g., at the University of Alabama at Birmingham and at Duke University; Ramirez-Zamora et al., 2020). The neural mechanisms that allow generation of the ERNA signal have yet to be established. One hypothesis is that ERNA could reflect the resonant state of neuronal networks that support the emergence of oscillatory activity in local field potentials. It is notable that ERNA and high frequency oscillations occupy a similar frequency band in STN recordings (Sinclair et al., 2019). Modeling studies have also indicated that ERNA could arise from interactions between the STN and the pallidum, due to quasi-periodic pallidal inhibition following DBS pulses (Ramirez-Zamora et al., 2020).

## Potential Clinical Applications of ERNA

ERNA may have utility to improve STN DBS in the following applications.

### Electrode Implantation Guidance

ERNA is reliably recordable during STN DBS implantation—our group has done so in over 175 STN's. Crucially, ERNA appears to localize to the STN and is absent from recordings from white matter tracts adjacent to the STN (e.g., the posterior subthalamic area) (Sinclair et al., 2018). ERNA amplitude also varies within the STN, being greatest in the dorsal subregion (Figures 1C,D). ERNA recordings during surgery would therefore seem to offer a distinct potential advantage to standard microelectrode recordings—which do not reliably localize the ideal STN target (Soares et al., 2019). A further substantial benefit of ERNA is that it is recordable under general anesthesia, using standard agents such as propofol, sevoflurane, and isoflurane (Sinclair et al., 2018, 2019b) (Figure 1E), which could enable accurate localization of the STN during asleep implantation surgery.

### DBS Programming

The localization of ERNA to the dorsal STN subregion raises the possibility that ERNA could serve as a biomarker to select beneficial electrode configurations to use for chronic DBS therapy. Indeed, in a recently completed, double-blinded, experimental study in 14 PD patients (28 STN's) performed in the off medication state (Sinclair et al., 2018; Xu et al., 2019), we found that electrodes with larger ERNA amplitudes produced greater clinical benefit from DBS (Figure 1F), and simply selecting the electrode with the greatest ERNA RMS amplitude over 4–20 ms yielded a hemibody motor benefit from DBS that approximated the maximal available benefit from each electrode array. The identification of beneficial electrode configurations may also be further enhanced by factoring in other ERNA features, such as frequency and decay rate. It can also be speculated that the localization of ERNA will result in variation around the different segments of steering electrodes, which may assist identification of segments to use for chronic therapy.

Our group is also investigating whether ERNA may contain information to help identify beneficial DBS frequencies to apply in individual patients. It is notable that ERNA frequency modulates with therapeutic DBS, with median frequencies across patients reducing from around 310 Hz to plateau around 260 Hz (Figure 1G)—two times the stimulation frequency applied (130 Hz), a rate that is considered to be effective in most patients. The exact frequency that ERNA decreases to varies across individuals, ranging from about 230–320 Hz, suggesting there are patient specific frequencies associated with therapeutic benefit. Thus, the clinical effectiveness of applying DBS at a subharmonic of the ERNA plateau frequency could be assessed as a potential method for “fitting” a DBS frequency tailored to the individual.

### Adaptive DBS

The finding that ERNA frequency modulates with DBS also raises the possibility that this parameter could be used as a feedback signal for adaptive DBS control. Currently, we have only limited evidence that such modulation of ERNA frequency relates to the therapeutic efficacy of DBS on Parkinsonism. For example, we have performed limited intraoperative clinical assessments during DBS whilst recording ERNA and found that relief of upper limb akinesia and rigidity coincides with DBS levels where ERNA frequency plateaus (Sinclair et al., 2019). Moreover, ERNA frequency was found to correlate with the amplitude of beta band activity (Figure 1H), which can be considered a reasonable surrogate marker of akinesia and rigidity (Kühn et al., 2008, 2009). ERNA amplitude was also found to be modulated by DBS, suggesting it may have a role as a feedback signal; however, care needs to be taken to distinguish therapeutic effects from changes due to altering the stimulus intensity, such as variation in the volume of activation and saturation of neural firing (Sinclair et al., 2019).

## Discussion

ERNA is an exciting new prospect for a feedback signal that could be used to address shortcomings in the clinical application of STN DBS therapy for Parkinson's disease. It has key features of a large amplitude, localization to dorsal STN, is modulated by therapeutically-effective DBS, and is reliably measurable across patients, including under general anesthesia. However, while these attributes suggest it has promise for a variety of clinical applications, further work is required to validate and realize the potential of ERNA. For instance, additional evidence is required to establish how ERNA parameters reflect patient state and pathology, the clinical relevance of their modulation, and their effectiveness as feedback signals for adaptive control. Moreover, while findings indicate that ERNA localizes to dorsal STN, further work is also required to assess the location sensitivity and specificity of ERNA, and to more precisely determine the STN region and circuits responsible for its generation. Regardless of whether the source of ERNA coincides with the “sweet spot” for STN DBS therapy for PD (Horn et al., 2017), accurate determination of the origin of ERNA will further enable its use as a robust landmark for guiding electrode implantation surgery and programming. Whether evoked potentials may assist in identifying other DBS targets, such as the pallidum, is also worth assessing.

There have been a number of recent innovations in DBS technology aimed at addressing the shortcomings of existing devices and techniques. These advances include the use of intraoperative imaging techniques to guide implantation surgery under general anesthesia (Starr et al., 2010; Burchiel et al., 2013; Ho et al., 2018; Yin et al., 2019), the introduction of directional electrodes (Contarino et al., 2014), the proof-of-concept of adaptive DBS (Rosin et al., 2011; Little et al., 2013; Arlotti et al., 2016; Swann et al., 2018), and development of implantable pulse generators with sensing capabilities to realize closed-loop control (Stanslaski et al., 2012; Neumann et al., 2016). The attributes of ERNA suggest that it could potentially augment and/or complement the application of these technologies. For instance, imaging-guided electrode implantation has been shown to have comparable outcomes to conventional awake surgery (Ho et al., 2018; Yin et al., 2019); however, using imaging alone places greater emphasis on anatomy, which may not fully reflect each individual's physiology. As such, an electrophysiological signal that is readily measurable under general anesthesia and localized to the neural target could be used in conjunction with intraoperative imaging to better identify beneficial electrode locations.

Furthermore, directional electrodes provide the ability to “steer” stimulation toward target neural structures and away from those that elicit side-effects, at the expense of increased programming complexity due to the larger number of possible electrode configurations. Spontaneous beta band activity has been shown to be potentially helpful at selecting directional segments to use for chronic therapy (Tinkhauser et al., 2018). While the variation in ERNA around directional electrode arrays needs further assessment, the localization of ERNA to dorsal STN suggests it could be used to guide programming in the same manner as beta. Beta band activity has also been proposed as a method to guide electrode implantation; however, it's intraoperative use can be limited by micro-lesioning effects caused by electrode insertion (Chen et al., 2006). In addition to providing a larger and more robust signal, ERNA may also provide complementary information to beta, as they occupy different frequency bands. The frequency of ERNA is around 200–500 Hz, which is consistent with the frequency of spontaneous high frequency oscillations that have been found to couple with beta activity and to be modulated by movement, medication, and DBS (Foffani et al., 2003; López-Azcárate et al., 2010; Özkurt et al., 2011; Sinclair et al., 2019). Thus, systems that use ERNA and beta activity, and/or other spontaneous or evoked measures, may ultimately prove to be most effective for automated programming and adaptive DBS applications.

## Author Contributions

WT, NS, KB, and HM contributed to the conception, drafting and revision of the manuscript, and read and approved the submitted version.

### Conflict of Interest

The authors are named inventors on patents relating to ERNA, which are assigned to DBS Technologies Pty Ltd. WT, NS, KB, and HM hold shares and/or options in DBS Technologies Pty Ltd.
